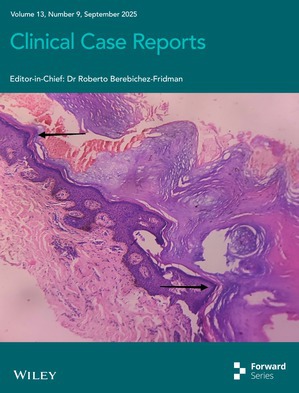# Cover Image

**DOI:** 10.1002/ccr3.70912

**Published:** 2025-09-24

**Authors:** Mahesh Mathur, Sumit Paudel, Sandhya Regmi, Sambidha Karki, Shilpa Maharjan, Nabita Bhattarai

## Abstract

The cover image is based on the article *“Clinical Overlap of Darier's Disease and Acrokeratosis Verruciformis of Hopf”: A Case Report* by Mahesh Mathur et al., https://doi.org/10.1002/ccr3.70862.